# Association between Depression, Lifestyles, Sleep Quality, and Sense of Coherence in a Population with Cardiovascular Risk

**DOI:** 10.3390/nu13020585

**Published:** 2021-02-10

**Authors:** Aina Riera-Sampol, Miquel Bennasar-Veny, Pedro Tauler, Mar Nafría, Miquel Colom, Antoni Aguilo

**Affiliations:** 1 Research Group on Evidence, Lifestyles and Health, Department of Nursing and Physiotherapy, Research Institute of Health Sciences (IUNICS), University of the Balearic Islands, 07122 Palma, Spain; ana.riera@uib.es (A.R.-S.); miquel.bennasar@uib.es (M.B.-V.); marfnafria@gmail.com (M.N.); miquelcolomrossello@gmail.com (M.C.); aaguilo@uib.es (A.A.); 2 Health Research Institute of the Balearic Islands (IdISBa), 07120 Palma, Spain; 3 Research Group on Evidence, Lifestyles and Health, Department of Fundamental Biology and Health Sciences, Research Institute of Health Sciences (IUNICS), University of the Balearic Islands, 07122 Palma, Spain

**Keywords:** depression, lifestyles, Mediterranean diet, physical activity, sleep quality, cardiovascular risk, sense of coherence

## Abstract

People with cardiovascular risk have more depression than the general population. Depression and cardiovascular risk have been commonly linked to lower sense of coherence (SOC) values, unhealthy lifestyles, and poor sleep quality. The aim of this study was to analyze the association between depression, health-related lifestyles, sleep quality, and SOC in a population with cardiovascular risk. A cross-sectional study was conducted in 310 participants (aged 35–75 years) with cardiovascular risk. Sociodemographic and anthropometric characteristics, cardiovascular risk, SOC score, depression levels, sleep quality, and lifestyles (physical activity, diet quality (measured as the adherence to the Mediterranean diet), and tobacco and alcohol consumption) were determined. The regression analysis showed significant associations between depression levels and sex (odds ratio (OR): 2.29; 95% CI: 1.29, 4.07), diet (OR: 0.85; 95% CI: 0.73, 0.99), body mass index (BMI) (OR: 1.06; 95% CI: 1.01, 1.12), cardiovascular disease (CVD) (OR: 2.55; 95% CI: 1.18, 5.48), sleep quality (OR: 0.26; 95% CI: 0.15, 0.46), and SOC (OR: 0.96; 95% CI: 0.94, 0.98). Protective effects of male sex, a lower BMI, no CVD, a higher adherence to the Mediterranean diet, a high sleep quality, and a higher SOC were found. In conclusion, among lifestyles determined, only diet was associated with depression levels. SOC and sleep quality were also found as significant predictors for depression levels.

## 1. Introduction

Depressive and anxiety disorders are the two main diagnostic categories of common mental disorders. In 2015, the global prevalence of depression was estimated to be 4.4%, with higher figures for females (5.1%) than for males (3.6%) and with higher prevalence rates in older adulthood [1]. The estimated number of people living with depression increased by 18.4% between 2005 and 2015 [2], and WHO has considered depression as the single largest contributor to global disability [1].

It has been reported that disordered sleep is strongly associated with depression [3,4,5,6]. Insomnia increases the risk of developing depression and negatively affects the disease’s trajectory, and it is associated with higher suicide rates [5]. Insomnia is considered an important symptom in depression diagnostics [6]. Depression disorders have also been linked to lifestyles, and the beneficial roles of a proper diet and physical activity levels and of limited alcohol and tobacco consumption have been reported [7,8,9]. When diet has been analyzed in relation to depression, the Mediterranean diet has been used as one of the main approaches, with some studies reporting its beneficial effect [10,11,12].

Salutogenesis is a new paradigm of positive health that focuses on protective health factors, in contrast to the traditional pathogenic approach. This approach was studied by Antonovsky, who developed the salutogenic approach, paying close attention to the Sense of Coherence (SOC), its main theoretical construct [13]. SOC postulates three psychological dimensions that allow one to face the vital stressors: comprehensibility (the ability to define life events as less stressful), manageability (mobilizing resources to deal with encountered stressors), and meaningfulness (having levels of experience, motivation, desire, and commitment that are necessary to cope) [14]. Therefore, SOC plays an important role in managing one’s own health behaviors and helps to mobilize forces to deal with stressors and manage stress successfully [13].

Interestingly, strong negative correlations have been found between SOC scores and measures of depression and anxiety in adults, and physiological health parameters correlate in a similar way to SOC and measures of anxiety and depression [15,16,17,18,19]. The SOC scale also seems to be a valid instrument and a resource for the promotion of health, strengthening resilience and facilitating a subjective and positive state of health [14]. SOC is believed to associate with a healthy lifestyle, fewer atherosclerotic risk factors, and lower risk of cardiovascular disease (CVD) and all-cause mortality [20,21]. Furthermore, it has been reported that subjects with a low SOC score reported more sleep problems than subjects with a high SOC score [22]. However, associations between depression, lifestyles, sleep quality, and SOC have not been previously analyzed.

The aim of this study was to analyze the association between depression, health-related lifestyles (diet, physical activity, smoking, and alcohol consumption), sleep quality, and SOC in a population with cardiovascular risk. This population was considered because it has been reported that patients with CVD risk experience more depression than the general population [20]. Furthermore, both depression and CVD have been associated with lower SOC values, unhealthy lifestyles, and poor sleep quality. The gender perspective and the educational level were also included in the analysis. We hypothesized that high levels of SOC, healthy lifestyles, and high sleep quality are associated with lower depression levels.

## 2. Materials and Methods

### 2.1. Study Design and Participants

The present analysis reports baseline data from the ACTIVOS study [23]. For the present study, a descriptive cross-sectional design was conducted in patients with cardiovascular risk factors from 20 primary care centers, including both urban and rural centers (6 urban and 14 rural) in Mallorca (Spain). All the participants were informed of the purpose and demands of the study before giving their written consent to participate. The protocol was in accordance with the Declaration of Helsinki for research with human participants and was approved by the Institutional Review Board of the Balearic Islands Health Service Research Ethics (CEI-IB Ref. No. 2341/14).

Inclusion criteria were: patients aged 35–75 years with at least two cardiovascular risk factors and a cardiovascular risk of up to 15% measured using the Framingham-REGICOR (Registre Gironí del Cor) equation. CVD risk factors considered were: a family history of CVD, age over 55 years in men or over 65 years in women, a smoking habit, hypertension, a presence of diabetes, dyslipidemia, and obesity (body mass index (BMI) > 30 kg/m^2^). Exclusion criteria were: institutionalization, Barthel index of <60, terminal illness, dementia or cognitive impairment, bypass or coronary angioplasty in the previous 3 months, unstable coronary heart disease or untreated heart failure, the presence of myocardial infarction, residence outside the health care area, and participation in another research study. Initially, 370 participants were recruited, but data could not be obtained from 60 of these participants, leading to a final sample of 310 participants (157 males and 153 females).

### 2.2. Data Collection

All the data were collected by well-trained nurses using previously validated standard procedures during an only visit of each participant to the Primary Health care center. The following data were collected:

Sociodemographic variables—age, sex, education level, and employment status (active, unemployed, or retired). Two categories for education level were considered: completion of primary education or less, and secondary education or university.

Health status. The self-assessment of health status was obtained by using the question “How do you rate your overall health status (within the last 12 months)?” Self-assessment of health status has been proven to be a good indicator of global health [24].

Anthropometric measurements. Body weight (electronic scale Seca 700; Seca, Hamburg, Germany) and height (Stadiometer Seca 220 CM Telescopic Height Rod for Column Scales, precision 0.5 cm; Seca, Hamburg, Germany) were measured, and BMI was calculated as weight (kg) divided by height (m) squared. Participants were categorized depending on their BMI following WHO criteria: underweight (BMI < 18.5 kg/m^2^), normal weight (BMI = 18.5 to 24.9 kg/m^2^), overweight (BMI = 25.0 to 29.9 kg/m^2^), and obese (BMI ≥ 30.0 kg/m^2^) [25].

Cardiovascular risk, cardiovascular risk factors and chronic diseases. Cardiovascular risk was determined using the Framingham-REGICOR score, a validated equation that entails a calibration of the Framingham score for the Spanish population. Participants were classified as having low (values lower than 5%), moderate (5–9.9%), or high cardiovascular risk (≥10%) [26]. The Framingham-REGICOR algorithm considers the following parameters: age, sex, smoking habit, the presence or absence of diabetes, blood total cholesterol and HDL (high-density lipoproteins) cholesterol levels, and systolic and diastolic blood pressure (BP). Serum concentrations of total cholesterol, HDL cholesterol, LDL (low-density lipoproteins) cholesterol, and triacylglycerides were measured using an autoanalyzer (SYNCHRON CXH9 PRO; Beckman Coulter, Brea, CA, USA). Systolic and diastolic BP was taken with an automatic and calibrated sphygmomanometer (OMRON M3; OMRON Healthcare Europe, Barcelona, Spain). Information about participants’ chronic diseases was obtained by each nurse from the Primary Health care center records.

Sense of coherence. The sense of coherence (SOC) was determined using the SOC-13 questionnaire, a short version that has been previously validated [27]. SOC values range from 13 to 91 and include three dimensions: comprehensibility, manageability and meaningfulness. When adequate, SOC was categorized into tertiles, as no cut-off points were validated.

Physical activity levels. The validated standard short form of the International Physical Activity Questionnaire (IPAQ) was used to determine participants’ physical activity performed in metabolic equivalent (METs) minutes a week [28]. Participants were also categorized in high, moderate, and low physical activity levels.

Diet quality was determined using an assessment of adherence to the Mediterranean diet, a 14-item questionnaire previously developed and validated for the Spanish population [29]. Each item is scored as 0 or 1, and a global score of 9 or higher indicates a good adherence to the Mediterranean diet.

Alcohol consumption. The consumption of alcohol beverages (yes/no), the frequency of alcohol consumption, and the number of consumptions per day were recorded. In addition, the total amount of ethanol (g) consumed per week was also determined.

Smoking habit. Participants were asked if they smoked and were then classified as a non-smoker, current smoker, or former smoker, according to WHO criteria.

Sleep quality was measured using the short form of the MOS (Medical Outcomes Study) Sleep Scale, a validated six-item self-report questionnaire [30]. The MOS Sleep Scale ranges from 6 to 30, and the higher the MOS Sleep Scale value is, the worse the sleep quality is. A high sleep quality was considered when the score obtained was lower than 11 points.

Depression. The Spanish validated version of the Patient Health Questionnaire (PHQ-9) was used [31]. The PHQ-9 does not suppose a screening tool for depression, but it is used to monitor the severity of depression and response to treatment, and it has been validated for primary care use [32]. Scores of 5, 10, 15, and 20 represent cut-off points for, respectively, low, moderate, severe, and very severe depression [33]. Scores lower than 5 were considered as no depression.

### 2.3. Statistical Analysis

Statistical analysis was carried out using IBM SPSS Statistics 24.0 software (SPSS/IBM, Chicago, IL, USA). Statistical significance was accepted at a *p*-value below 0.05. All the data were tested for their normal distribution (Kolmogorov–Smirnov test). Descriptive analysis was used to report the frequencies and percentages of categorical variables, and the mean and standard deviation (SD) were reported for quantitative variables. A Student’s *t*-test for unpaired data or Pearson’s chi-square (χ^2^) test was used to evaluate differences between sexes. Listwise deletion (complete case) was used for handling missing data. A Student’s *t*-test for unpaired data, or a one-way ANOVA, was used to determine differences in depression levels in participants categorized per: SOC (tertiles), Mediterranean diet (low and high adherence), physical activity (low, moderate and high levels), alcohol consumption (yes/no), smoking habit (current, former and no smokers), sleep quality (low and high), BMI (normal, overweight and obese) and CVD (yes/no).

To evaluate the association of different risk factors with depression, an adjusted logistic regression model was used. Depression risk factors considered were sex, age, BMI, education level, comorbidities (dyslipidemia, hypertension, diabetes and CVD), SOC, Mediterranean diet score, physical activity, smoking habit, alcohol consumption and sleep quality. All variables that were considered relevant and those with a *p*-value below 0.25 in the univariate analyses were included in the adjusted model. Each variable was extracted (one-by-one) according to the level of significance in the model. Possible interactions and their statistical significance were evaluated, and possible collinearity was determined. The goodness of fit of the model was assessed via calculation of the area under the curve (receiver operating characteristic, ROC curve) and the Hosmer–Lemeshow C statistic [34].

## 3. Results

### 3.1. General Characteristics of Participants

Table 1 shows the general characteristics of participants in the study as a whole and categorized per sex. Most participants had high BMI values and were retired. Female participants presented lower values of cardiovascular risk (CVR), waist circumference, triglycerides and diabetes prevalence, but higher values of HDL cholesterol.

### 3.2. Health-Related Lifestyles, SOC, Sleep Quality, and Depression Levels of Participants

Table 2 shows values for participants’ lifestyles, SOC, depression, and sleep quality. Male participants reported better health status, a higher SOC value, a higher percentage of alcohol consumers, a higher percentage of smokers, a higher cardiovascular risk, and a longer time performing physical activity. On the other hand, female participants showed higher depression levels, with higher percentages of moderate and high depression than males. Regarding adherence to the Mediterranean diet, no differences between sexes were observed.

Table 3 shows depression values in participants categorized by several independent variables. Significant differences were found between categories for SOC (*p* < 0.001), adherence to the Mediterranean diet (*p* = 0.029), physical activity (*p* = 0.016), and sleep quality (*p* < 0.001). Lower depression values were found for high adherence to the Mediterranean diet and for high sleep quality. Participants with moderate levels of physical activity showed lower depression levels than participants with low levels of physical activity (*p* = 0.018). Lower depression values were found for Tertile 3 of SOC with respect to Tertile 2 (*p* < 0.001) and to Tertile 1 (*p* < 0.001).

### 3.3. Logistic Regression Model for Depression

Table 4 shows the factors associated with depression. Univariate analyses show that women, those who had higher BMI, those with CVD, those who had lower SOC, those who had bad sleep quality and a low adherence to the Mediterranean diet had greater depression. Multivariate analyses, which controlled for all these factors indicated that women, presence of CVD, higher BMI, lower SOC, and poor sleep quality remained significantly associated with depression. ROC analysis showed that a model that considered age, sex, SOC, Mediterranean diet, and sleep quality had a high predictive capacity of depression (AUC = 0.817, 95% CI 0.768 to 0.866). A sensitivity analysis adjusting for comorbidities was also performed (data not shown), reporting results very similar to the reported ones.

## 4. Discussion

The main finding of the present study was that, in a population with cardiovascular risk, lower depression levels were associated with a higher SOC, a high sleep quality, being male, a lower BMI, absence of CVD, and a higher adherence to the Mediterranean diet. However, no associations were found between depression levels and lifestyles such as a smoking habit, alcohol consumption, and physical activity levels. It is noteworthy that participants in the present study showed low levels of depression, with an average just over the cut-off point for low levels of depression. Within these low levels, the common differences between sexes [1,2] were also observed in the present study, with values for males under this low cut-off point and values for females over this limit.

Diet has been considered a modifiable risk target to reduce the prevalence of depression. Diet, as well as other lifestyles, could influence some pathways and imbalances associated with depression such as neurotransmitter imbalances, hypothalamic–pituitary–adrenal axis disturbances, dysregulated inflammatory pathways, increased oxidative damage, neuroprogression, and mitochondrial disturbances [9]. Therefore, the association between the quality of the diet, measured as the adherence to the Mediterranean diet model, and depression was ascertained in the present study. In this regard, an association between adherence to the Mediterranean diet and the absence of depression has been reported [11,35]. In the present study, a beneficial effect of adherence to the Mediterranean diet was also observed, as a significant protective effect of a higher adherence was found. Because associations between higher SOC values and healthy lifestyles have commonly been reported [21], it is striking that the association found in the present study between depression and diet was independent of SOC levels, highlighting the direct association between depression and Mediterranean diet.

One of the main characteristics of the Mediterranean diet is a high intake of fish, vegetables, and fruit. Some nutrients found in these food groups have been shown to play key roles in depression [11]. In this sense, it has been shown that the intake of n-3 polyunsaturated fatty acids (n-3 PUFAs) such as docosahexaenoic acid (DHA), eicosapentaenoic acid (EPA), and alpha-linolenic acid (ALA), which are known to be present mainly in fish and vegetable oils, have a beneficial effect on depression [12,36]. The protective effects of the Mediterranean diet have also been attributed to the intake of fruits and vegetables [37]. Fruits and vegetables are important sources of specific nutrients, suggested to exert beneficious effects on mental health such as complex carbohydrates and fiber, vitamin C, B vitamins, carotenes, potassium, and polyphenols [38]. Some of these nutrients (vitamin C, carotenes, and polyphenols) are important antioxidants, and increased oxidative stress and deficient antioxidant levels have been found in patients with major depression [9]. However, it cannot be ignored that a higher level of mental health promotes better diet, including higher fruit and vegetable intake [39]. In the present study, the association between an adequate intake of fruit and vegetable and depression levels could not be properly analyzed because only 31 participants reported an intake of three or more fruit servings per day, and only eight participants reported a daily intake of at least three vegetable servings. This observation could reinforce the suggestion that the variation of the dietary components of the Mediterranean diet could be the main reasons for its protective role against depression [11,36,37]. The protective effect of the Mediterranean diet has also been associated with the intake of dairy products such as high-fat yogurts [40], another food group with a high intake in this diet. However, the Mediterranean diet is only one of the characteristics of the Mediterranean lifestyle, characterized by proper rest, adequate physical activity levels, conviviality, etc. All these facts suggest that the overall lifestyle could have protective effects that are not only linked to specific nutrients or food groups, but also to psychological, social, and physical behaviors [41].

The presence of mental illness has commonly been associated with having not only poorer diet but also other poorer lifestyles [42]. In the present study, the association between depression levels and other lifestyles such as physical activity, smoking, and alcohol consumption was also analyzed. However, no association between these lifestyles and depression was found. This lack of association could be explained by certain characteristics of the sample. Regarding physical activity and depression, the lack of association observed in the present study could be related to the very low levels of depression observed, as it has been reported that anti-depressive effects of physical activity are weaker in non-clinical populations than in clinical populations presenting severe depression [43]. Furthermore, most of the participants present adequate physical activity levels, achieving the minimum recommendations of the WHO [44] and, probably, the health effects of being active. However, it is noteworthy that an unhealthy association between depression levels and BMI, with higher depression levels for increased BMI values was observed. This association has been previously described, and it has been suggested to be bidirectional, with a broad range of factors involved [45]. Furthermore, it should be considered that, in the present study, the association between BMI and depression was independent of the SOC, as it was for diet. It is worth mentioning that this association was found in a population with BMI values mostly belonging to the overweight and obese categories. Therefore, it seems that, even within these unhealthy BMI values, depression levels increase with BMI values. This observation is in line with the conclusion of a previous review reporting an increased risk of depression as BMI increases, with worse depression figures for obesity than for overweight, and with a higher risk as the severity of obesity increases [45].

Previous studies have suggested a very strong link between depression and sleep disturbance [3,4,5,6]. In depressed subjects, sleep continuity is often impaired, sleep efficiency decreased, and total sleep time reduced [6]. The results from the present study agree with an association between poor sleep quality and increased depression levels. The mechanistic relation between depression and sleep is not fully understood, and it has been considered to be complex and likely bidirectional [4]. However, despite this observation, it has been stated that sleep problems often precede depression [3]. In this sense, it has been suggested that “feeling well rested” due to adequate and better sleep could lead to a more energetic self, capable of reaching personal goals and being socially connected with others [46].

In agreement with previous results [15,16], higher levels of depression were associated with lower SOC scores. Haukkala et al. [20] also reported this association in patients with cardiovascular disease, showing that the positive construct of SOC has independent predictive power over and beyond the depressive. It has been suggested that the common associations found between depression, or depression symptoms, and SOC might result in an overlap in their associations with, among others, adverse disease and mortality outcomes [20]. In this regard, it has been suggested that the SOC scale could be an inverse measure of persistent depressive symptoms and, according to a study in adolescent women, these symptoms were even better captured by SOC than by specialized scales for anxiety and depression [47]. Following this suggestion, SOC could be used to make a tentative diagnosis of depression in at-risk populations. On the other hand, as Milaniak et al. [19] suggested for patients after heart transplantation, the intervention focused on personal resources could be useful to improve psychological outcomes in patients with cardiovascular risk. Therefore, SOC could be applied as a positive resource for combating depression [48].

This study presents some limitations that should be acknowledged. The observational and cross-sectional nature of the study does not allow for the establishment of any causal association between dependent and independent variables. Though well-known validated questionnaires were used, independent and some dependent variables such as physical activity and diet quality were self-reported. It is also possible that unmeasured lifestyle or demographic variables confounded the observed relationships. Furthermore, the relatively small sample size may have limited statistical power and therefore precluded additional significant observations. Some characteristics of the sample, such as the lower number of participants with normal BMI or with a proper intake of fruit and vegetables, could also have contributed to limited significant observations.

## 5. Conclusions

While a proper diet, characterized by a higher adherence to the Mediterranean diet, was associated with lower depression levels, other important lifestyles such as a smoking habit, alcohol consumption, and physical activity levels did not show any association with depression. Sleep quality and SOC score were also associated with depression levels, with a protective effect of a higher SOC and a high sleep quality. It is noteworthy that the association between depression levels and diet, as well as with BMI, were independent of SOC. The relationship between the SOC and depression allows us to highlight the importance of the salutogenic approach as a good strategy against depression. Future studies should consider the implementation of interventions aiming to increase the SOC.

## Figures and Tables

**Table 1:** General characteristics of participants in the study.

	All (*n* = 310)	Men (*n* = 157)	Women (*n* = 153)	*p*-Value
	Mean (SD) or *n* (%)			
**Age (years)**	62.2 (8.8)	62.2 (8.8)	62.2 (8.7)	0.979
**Education level**				
Primary or less	161 (51.9%)	80 (51.0%)	81 (52.9%)	0.726
Secondary or high	149 (48.1%)	77 (49.0%)	72 (47.1%)	
**Employment status**				0.021
Active	94 (31.2%)	58 (37.2%)	36 (24.8%)	
Unemployed	34 (11.3%)	12 (7.7%)	22 (15.2%)	
Retired	173 (57.5%)	86 (55.1%)	87 (60.0%)	
**Height (cm)**	162.6 (9.1)	168.5 (6.8)	156.7 (7.1)	<0.001
**Weight (kg)**	83.1 (16.8)	87.9 (7.0)	78.2 (7.1)	<0.001
**BMI (kg/m²)**	31.4 (5.6)	30.9 (5.2)	31.8 (5.9)	0.120
**BMI classification**				0.616
Normal weight	26 (8.4%)	14 (9.0%)	12 (7.8%)	
Overweight	116 (37.6%)	62 (39.7%)	54 (35.3%)	
Obese	167 (54.0%)	80 (51.3%)	87 (56.9%)	
**Total cholesterol (mg/dL)**	199.7 (111.4)	198.2 (108.6)	201.2 (37.6)	0.815
**LDL cholesterol (mg/dL)**	114.4 (138.2)	108.6 (39.0)	131.3 (61.7)	0.029
**HDL cholesterol (mg/dL)**	53.3 (23.9)	48.9 (21.1)	57.8 (25.8)	0.001
**TG (mg/dL)**	140.5 (78.6)	149.4 (91.5)	131.3 (61.7)	0.046
**SBP (mm Hg)**	131.8 (16.9)	133.3 (18.2)	130.3 (15.4)	0.119
**DBP (mm Hg)**	79.1 (8.9)	79.1 (9.6)	79.0 (8.2)	0.932
CVR *	6.4 (4.0)	7.7 (4.5)	5.1 (2.7)	<0.001
**CVR classification**				
Low Risk	81 (31.8%)	21 (16.4%)	60 (47.2%)	
Moderate Risk	123 (48.2%)	71 (55.5%)	52 (40.9%)	
High Risk	51 (20.0%)	36 (28.1%)	15 (11.8%)	
**Dyslipidaemia (Yes)**	227 (73.2%)	116 (73.9%)	111 (72.5%)	0.790
**Hypertension (Yes)**	246 (79.4%)	128 (81.5%)	118 (77.1%)	0.338
**Diabetes (Yes)**	168 (54.2%)	100 (63.7%)	68 (44.4%)	0.001
**CVD (Yes)**	56 (18.1%)	32 (20.4%)	24 (15.7%)	0.283

* Determined using the Framingham-REGICOR scale; BMI: Body Mass Index; LDL: low-density lipoproteins; HDL: high-density lipoproteins; TG: Triacylglycerides; SBP: Systolic Blood Pressure; DBP: Diastolic Blood Pressure; CVR: Cardiovascular Risk; CVD: Cardiovascular disease (includes ischemic heart disease, stroke and heart failure). *p*-value indicates the significance of the difference between sexes.

**Table 2:** Health-related lifestyles, sleep quality, and depression levels of participants.

	All (*n* = 310)	Men (*n* = 157)	Women (*n* = 153)	*p*-Value
	Mean (SD) or *n* (%)			
**Health status**				
High or very high	197 (63.8%)	112 (71.8%)	87 (55.6%)	0.003
SOC	68.8 (13.1)	72.5 (12.0)	65.1 (13.2)	<0.001
**SOC components**				
Comprehensibility	25.0 (6.5)	26.8 (6.0)	23.3 (6.4)	<0.001
Manageability	21.4 (5.0)	22.6 (4.3)	20.3 (5.3)	<0.001
Meaningfulness	22.3 (4.3)	23.1 (3.9)	21.5 (4.5)	0.001
**Mediterranean diet**				
Index	8.82 (2.0)	8.78 (2.0)	8.87 (2.0)	<0.001
Low adherence	119 (40.2%)	60 (40.3%)	59 (40.1%)	
High adherence	177 (59.8%)	89 (59.7%)	88 (59.9%)	
**Physical activity**				
METs·minute/week	1818.7 (1823.9)	2082.4 (2006.9)	1546.3 (15,473.9)	0.010
Low levels	79 (25.5%)	38 (24.2%)	41 (26.8%)	
Moderate levels	193 (62.3%)	91 (58.0%)	102 (66.7%)	
High levels	38 (12.2%)	28 (17.8%)	10 (6.5%)	
**Alcohol consumption**				
Yes	105 (35.0%)	82 (54.3%)	23 (15.4%)	<0.001
Grams/day	8.43 (18.5)	14.26 (23.7)	1.88 (4.4)	<0.001
**Smoking habit**				<0.001
Current smoker	57 (18.8%)	30 (19.6%)	27 (17.9%)	
Former smoker	122 (40.1%)	85 (55.6%)	37 (24.5%)	
**Sleep quality**				
MOS Sleep Score	12.0 (4.6)	11.0 (4.4)	13.1 (4.6)	<0.001
High quality	146 (47.2%)	90 (57.7%)	56 (36.6%)	
**Depression**				
PHQ-9 score	5.1 (4.5)	3.8 (3.9)	6.4 (4.8)	<0.001
Low	194 (62.8%)	121 (77.6%)	73 (47.7%)	
Moderate	74 (23.9%)	22 (14.1%)	52 (34.0%)	
Severe or very severe	41 (13.3%)	13 (8.3%)	28 (18.3%)	

SOC: Sense of Coherence; MET: metabolic equivalent; MOS: Medical Outcomes Study; PHQ: Patient Health Questionnaire; *p*-value indicates the significance of the difference between sexes.

**Table 3:** Depression levels of participants in the study categorized by independent variables.

	Categories	Depression Levels (Mean (SD))	*p* Value
**SOC**	Tertile 1 (low SOC)	7.37 (4.79)	<0.001
	Tertile 2	5.00 (4.35)	
	Tertile 3 (high SOC)	2.75 (3.16)	
**Mediterranean diet**	Low adherence	5.84 (4.69)	0.029
	High adherence	4.66 (4.40)	
**Physical activity**	Low levels	6.32 (5.01)	0.016
	Moderate levels	4.66 (4.30)	
	High levels	4.33 (4.36)	
**Alcohol consumption**	Yes	4.51 (4.31)	0.113
	No	5.38 (4.64)	
**Smoking habit**	Current smoker	5.44 (5.04)	0.515
	Former smoker	4.72 (4.55)	
	No smoker	5.27 (4.34)	
**Sleep quality**	Low quality	6.95 (4.58)	<0.001
	High quality	2.97 (3.47)	
**BMI**	Normal weight	4.62 (4.73)	0.103
	Overweight	4.42 (4.64)	
	Obese	5.56 (4.42)	
**CVD**	Yes	6.21 (4.80)	0.035
	No	4.80 (4.45)	

SOC: Sense of Coherence; BMI: body mass index; CVD: cardiovascular disease (includes ischemic heart disease, stroke and heart failure); *p*-value indicates significant differences in depression levels between categories (Student’s *t*-test for unpaired data or one-way ANOVA).

**Table 4:** Univariate and multivariate analyses of independent variables associated with depression.

Depression	OR	95% CI	aOR	95% CI
**Sex**				
Female	3.129 ***	1.960–4.995	2.291 **	1.291–4.068
**Age (years)**	0.989	0.964–1.015	0.997	0.964–1.032
**BMI (kg/m^2^)**	1.067 **	1.023–1.112	1.064 *	1.007–1.124
**Education level**				
Primary or less	1.275	0.813–2.001		
**Dyslipidemia (Yes)**	1.128	0.679–1.876		
**Hypertension (Yes)**	1.030	0.592–1.794		
**Diabetes (Yes)**	0.998	0.636–1.565		
**CVD (Yes)**	2.056 *	1.141–3.702	2.547 *	1.184–5.480
**SOC**	0.935 ***	0.916–0.955	0.957 ***	0.934–0.980
**Mediterranean diet score**	0.818 **	0.726–0.922	0.850 *	0.731–0.989
**Physical activity**				
Moderate	0.583	0.331–1.027		
High	0.291	0.291–1.365		
**Smoking habit**				
No	0.964	0.541–1.720		
**Alcohol consumption**				
No	1.368	0.845–2.213		
**Sleep quality**				
High	0.163 ***	0.098–0.269	0.260 ***	0.146–0.463

*n* = 286; OR: odds ratio; CI: interval of confidence; aOR: adjusted odd ratio; SOC: Sense of Coherence; BMI: body mass index; CVD: Cardiovascular disease (includes ischemic heart disease, stroke and heart failure); * *p* < 0.05, ** *p* < 0.01, *** *p* < 0.001.

## Data Availability

The data presented in this study are available on request from the corresponding author. The data are not publicly available due to availability restrictions reported in the informed consent signed by all participants.

## References

[1] World Health Organization (2017). Depression and Other Common Mental Disorders Global Health Estimates.

[2] Vos T., Allen C., Arora M., Barber R.M., Brown A., Carter A., Casey D.C., Charlson F.J., Chen A.Z., Coggeshall M. (2016). Global, regional, and national incidence, prevalence, and years lived with disability for 310 diseases and injuries, 1990–2015: A systematic analysis for the Global Burden of Disease Study 2015. Lancet.

[3] McRae K., Gross J.J. (2020). Emotion regulation. Emotion.

[4] Kahn M., Sheppes G., Sadeh A. (2013). Sleep and emotions: Bidirectional links and underlying mechanisms. Int. J. Psychophysiol..

[5] Goldstein A.N., Walker M.P. (2014). The role of sleep in emotional brain function. Annu. Rev. Clin. Psychol..

[6] Nutt D., Wilson S., Paterson L. (2008). Sleep disorders as core symptoms of depression. Dialogues Clin. Neurosci..

[7] Sarris J., O’Neil A., Coulson C.E., Schweitzer I., Berk M. (2014). Lifestyle medicine for depression. BMC Psychiatry.

[8] Ruiz-Estigarribia L., Martínez-González M.Á., Díaz-Gutiérrez J., Sánchez-Villegas A., Lahortiga-Ramos F., Bes-Rastrollo M. (2019). Lifestyles and the risk of depression in the “Seguimiento Universidad de Navarra” cohort. Eur. Psychiatry.

[9] Lopresti A.L., Hood S.D., Drummond P.D. (2013). A review of lifestyle factors that contribute to important pathways associated with major depression: Diet, sleep and exercise. J. Affect. Disord..

[10] Trichopoulou A., Costacou T., Bamia C., Trichopoulos D. (2003). Adherence to a Mediterranean Diet and Survival in a Greek Population. N. Engl. J. Med..

[11] Masana M.F., Haro J.M., Mariolis A., Piscopo S., Valacchi G., Bountziouka V., Anastasiou F., Zeimbekis A., Tyrovola D., Gotsis E. (2018). Mediterranean diet and depression among older individuals: The multinational MEDIS study. Exp. Gerontol..

[12] Parletta N., Zarnowiecki D., Cho J., Wilson A., Bogomolova S., Villani A., Itsiopoulos C., Niyonsenga T., Blunden S., Meyer B. (2019). A Mediterranean-style dietary intervention supplemented with fish oil improves diet quality and mental health in people with depression: A randomized controlled trial (HELFIMED). Nutr. Neurosci..

[13] Antonovsky A. (1996). The salutogenic model as a theory to guide health promotion. Health Promot. Int..

[14] Eriksson M., Lindström B. (2006). Antonovsky’s sense of coherence scale and the relation with health: A systematic review. J. Epidemiol. Community Health.

[15] Guo L.-N., Liu Y.-J., McCallum J., Söderhamn U., Ding X.-F., Yv S.-Y., Zhu Y.-R., Guo Y.-R. (2018). Perceived stress and depression amongst older stroke patients: Sense of coherence as a mediator?. Arch. Gerontol. Geriatr..

[16] Wang Q., Hay M., Clarke D., Menahem S. (2014). Associations between knowledge of disease, depression and anxiety, social support, sense of coherence and optimism with health-related quality of life in an ambulatory sample of adolescents with heart disease. Cardiol. Young.

[17] López-Martínez C., Frías-Osuna A., Del-Pino-Casado R. (2019). Sense of coherence and subjective overload, anxiety and depression in caregivers of elderly relatives. Gac. Sanit..

[18] Möllerberg M.L., Årestedt K., Swahnberg K., Benzein E., Sandgren A. (2019). Family sense of coherence and its associations with hope, anxiety and symptoms of depression in persons with cancer in palliative phase and their family members: A cross-sectional study. Palliat. Med..

[19] Milaniak I., Wilczek-Rużyczka E., Wierzbicki K., Sadowski J., Kapelak B., Przybyłowski P. (2016). Role of Personal Resources in Depression and Stress in Heart Transplant Recipients. Transplant. Proc..

[20] Haukkala A., Konttinen H., Lehto E., Uutela A., Kawachi I., Laatikainen T. (2013). Sense of coherence, depressive symptoms, cardiovascular diseases, and all-cause mortality. Psychosom. Med..

[21] Geulayov G., Drory Y., Novikov I., Dankner R. (2015). Sense of coherence and 22-year all-cause mortality in adult men. J. Psychosom. Res..

[22] Hansen Å.M., Grynderup M.B., Rugulies R., Conway P.M., Garde A.H., Török E., Mikkelsen E.G., Persson R., Hogh A. (2018). A cohort study on self-reported role stressors at work and poor sleep: Does sense of coherence moderate or mediate the associations?. Int. Arch. Occup. Environ. Health.

[23] Riera-Sampol A., Bennasar-Veny M., Tauler P., Aguilo A. (2020). Effectiveness of physical activity prescription by primary care nurses using health assets: A randomized controlled trial. J. Adv. Nurs..

[24] Manor O., Matthews S., Power C. (2001). Self-rated health and limiting longstanding illness: Inter-relationships with morbidity in early adulthood. Int. J. Epidemiol..

[25] World Health Organization (2000). Obesity: Preventing and managing the global epidemic. Report of a WHO consultation. World Health Organ. Technol. Rep. Ser..

[26] Marrugat J., Vila J., Baena-Díez J.M., Grau M., Sala J., Ramos R., Subirana I., Fitó M., Elosua R. (2011). Validez relativa de la estimación del riesgo cardiovascular a 10 años en una cohorte poblacional del estudio REGICOR. Rev. Esp. Cardiol..

[27] Alsén P., Eriksson M. (2016). Illness perceptions of fatigue and the association with sense of coherence and stress in patients one year after myocardial infarction. J. Clin. Nurs..

[28] Craig C.L., Marshall A.L., Sjöström M., Bauman A.E., Booth M.L., Ainsworth B.E., Pratt M., Ekelund U., Yngve A., Sallis J.F. (2003). International physical activity questionnaire: 12-country reliability and validity. Med. Sci. Sports Exerc..

[29] Estruch R., Ros E., Salas-Salvadó J., Covas M.-I., Corella D., Arós F., Gómez-Gracia E., Ruiz-Gutiérrez V., Fiol M., Lapetra J. (2018). Primary Prevention of Cardiovascular Disease with a Mediterranean Diet Supplemented with Extra-Virgin Olive Oil or Nuts. N. Engl. J. Med..

[30] Kim S.S., Won J.C., Kwon H.S., Kim C.H., Lee J.H., Park T.S., Ko K.S., Cha B.Y. (2013). Validity of the medical outcomes study sleep scale in patients with painful diabetic peripheral neuropathy in Korea. J. Diabetes Investig..

[31] Diez-Quevedo C., Rangil T., Sanchez-Planell L., Kroenke K., Spitzer R.L. (2001). Validation and utility of the patient health questionnaire in diagnosing mental disorders in 1003 general hospital Spanish inpatients. Psychosom. Med..

[32] Haddad M., Walters P., Phillips R., Tsakok J., Williams P., Mann A., Tylee A. (2013). Detecting Depression in Patients with Coronary Heart Disease: A Diagnostic Evaluation of the PHQ-9 and HADS-D in Primary Care, Findings From the UPBEAT-UK Study. PLoS ONE.

[33] Kroenke K., Spitzer R.L., Williams J.B.W. (2001). The PHQ-9. J. Gen. Intern. Med..

[34] Hosmer D.W., Lemeshow S. (2000). Applied Logistic Regression.

[35] Sánchez-Villegas A., Toledo E., De Irala J., Ruiz-Canela M., Pla-Vidal J., Martínez-González M.A. (2012). Fast-food and commercial baked goods consumption and the risk of depression. Public Health Nutr..

[36] Grosso G., Micek A., Marventano S., Castellano S., Mistretta A., Pajak A., Galvano F. (2016). Dietary n-3 PUFA, fish consumption and depression: A systematic review and meta-analysis of observational studies. J. Affect. Disord..

[37] Liu X., Yan Y., Li F., Zhang D. (2016). Fruit and vegetable consumption and the risk of depression: A meta-analysis. Nutrition.

[38] Głąbska D., Guzek D., Groele B., Gutkowska K. (2020). Fruit and vegetable intake and mental health in adults: A systematic review. Nutrients.

[39] Lang U.E., Beglinger C., Schweinfurth N., Walter M., Borgwardt S. (2015). Nutritional aspects of depression. Cell. Physiol. Biochem..

[40] Perez-Cornago A., Sanchez-Villegas A., Bes-Rastrollo M., Gea A., Molero P., Lahortiga-Ramos F., Martínez-Gonźalez M.A. (2016). Intake of high-fat yogurt, but not of low-fat yogurt or prebiotics, is related to lower risk of depression in women of the SUN cohort study. J. Nutr..

[41] Bach-Faig A., Berry E.M., Lairon D., Reguant J., Trichopoulou A., Dernini S., Medina F.X., Battino M., Belahsen R., Miranda G. (2011). Mediterranean diet pyramid today. Science and cultural updates. Public Health Nutr..

[42] Parletta N., Aljeesh Y., Baune B.T. (2016). Health behaviors, knowledge, life satisfaction, and wellbeing in people with mental illness across four countries and comparisons with normative sample. Front. Psychiatry.

[43] Rebar A.L., Stanton R., Geard D., Short C., Duncan M.J., Vandelanotte C. (2015). A meta-meta-analysis of the effect of physical activity on depression and anxiety in non-clinical adult populations. Health Psychol. Rev..

[44] Bull F., Saad Al-Ansari S., Biddle S., Borodulin K., Buman M., Cardon G., Carty C., Chaput J.-P., Chastin S., Chou R. (2020). World Health Organization 2020 Guidelines on Physical Activity and Sedentary Behaviour. Br. J. Sports Med..

[45] Preiss K., Brennan L., Clarke D. (2013). A systematic review of variables associated with the relationship between obesity and depression. Obes. Rev..

[46] Hamilton N.A., Nelson C.A., Stevens N., Kitzman H. (2007). Sleep and psychological well-being. Soc. Indic. Res..

[47] Henje Blom E.C., Serlachius E., Larsson J.-O., Theorell T., Ingvar M. (2010). Low Sense of Coherence (SOC) is a mirror of general anxiety and persistent depressive symptoms in adolescent girls-a cross-sectional study of a clinical and a non-clinical cohort. Health Qual. Life Outcomes.

[48] Li W., Leonhart R., Schaefert R., Zhao X., Zhang L., Wei J., Yang J., Wirsching M., Larisch A., Fritzsche K. (2015). Sense of coherence contributes to physical and mental health in general hospital patients in China. Psychol. Health Med..

